# Expanding the Phenotypic Spectrum of DICER1 Syndrome: Case Report with Overgrowth, Macrocephaly, Multinodular Goiter, and Lung Cyst

**DOI:** 10.3390/children13070904

**Published:** 2026-07-08

**Authors:** Yuliya Bazdarska, Marek Niedziela, Violeta Iotova

**Affiliations:** 1 Department of Pediatrics, Medical University, 9002 Varna, Bulgaria; 2 Department of Pediatric Endocrinology and Rheumatology, Institute of Pediatrics, Karol Jonscher Clinical Hospital, 60-572 Poznan, Poland; marek.niedziela@ump.edu.pl; 3 Department of Pediatric Endocrinology and Rheumatology, Faculty of Medical Sciences, University of Medical Science, 60-512 Poznan, Poland

**Keywords:** DICER1 syndrome, GLOW syndrome, overgrowth, rare disease

## Abstract

**Highlights:**

**What are the main findings?**

- The initial clinical findings (overgrowth, developmental delay and macrocephaly) are not classic hallmarks of DICER1 syndrome.
- No clinical and radiology evidence of malignancy in proband and mother.

**What are the implications of the main findings?**

- Consider genetic testing in patients with overgrowth and positive family history for DICER1 variants.
- It underlines the variable expressivity of DICER1 syndrome and the fact that some of individuals remain asymptomatic. Highlighting the importance of the need for long-term follow-up.

**Abstract:**

**Background:** DICER1 syndrome is an autosomal dominant disorder with reduced penetrance and variable expressivity, predisposing carriers to a broad spectrum of benign and malignant tumors as well as non-neoplastic features. Recent reports suggest an expanded phenotype overlapping with GLOW syndrome, characterized by developmental anomalies in addition to tumor risk. **Case Presentation:** We describe a 16-year-old male with overgrowth, macrocephaly, developmental delay, multinodular goiter, and a unilateral lung cyst. Genetic testing identified a germline heterozygous nonsense variant in DICER1 (c.1525C>T, p.Arg509Ter), confirmed also in the patient’s mother. Family history included thyroid nodules, uterine tumor, and multiple malignancies on both maternal and paternal sides. The proband has remained free of neoplasia to date, and his lung cyst is stable under annual imaging surveillance. **Discussion:** The presented patient highlights the expanding phenotypic spectrum of DICER1-associated conditions, with features overlapping with GLOW syndrome but without Wilms tumor or cystic nephroma. The absence of malignancy in both the proband and his mother underscores the reduced penetrance and variable expressivity of DICER1 variants. Our findings reinforce the importance of structured surveillance, individualized risk assessment, and cautious management of asymptomatic carriers. **Conclusions:** This report expands the clinical spectrum of DICER1 syndrome, supporting its recognition as both a tumor predisposition and developmental disorder. Longitudinal studies are required to refine surveillance strategies and better define genotype–phenotype correlations.

## 1. Introduction

The DICER1 gene, located on chromosome 14q32.13, encodes a 1922–amino acid protein composed of 27 exons. It is highly conserved across species, reflecting its fundamental biological role. The DICER1 protein contains key functional domains, including two double-stranded RNA-binding domains and RNase IIIa and RNase IIIb domains, which are essential for the processing of double-stranded RNA. DICER1 plays a central role in microRNA biogenesis and is critical for post-transcriptional gene regulation, embryonic development, tissue differentiation, and cellular homeostasis. To date, more than 150 germline pathogenic variants have been reported and are cataloged in public databases such as the Leiden Open Variation Database and the Human Variome Project [1,2,3].

**DICER1 syndrome** is an autosomal dominant hereditary tumor predisposition disorder characterized by reduced penetrance and variable expressivity, meaning that not all individuals who carry a pathogenic variant will manifest clinical disease, and those who do may present with widely differing phenotypes. Affected individuals are predisposed to develop a broad spectrum of benign and malignant neoplasms, the most commonly encountered being pleuropulmonary blastoma (PPB), cystic nephroma, Sertoli–Leydig cell tumor (SLCT) of the ovary and multinodular goiter (MNG) [1,4]. The underlying process of tumorigenesis in DICER1 syndrome typically conforms to a “two-hit” model, in which an inherited or de novo germline loss-of-function mutation constitutes the first hit, while the second hit arises somatically and most often targets hotspot residues within the RNase IIIb domain of the DICER1 protein. This biallelic inactivation disrupts the normal biogenesis of microRNAs, thereby altering gene expression networks in a manner that promotes oncogenesis [5]. While the majority of pathogenic DICER1 variants are inherited from an affected parent, between 10% and 20% of cases arise due to de novo mutations [6].

In addition to tumor predisposition, DICER1 syndrome is associated with non-neoplastic manifestations, most commonly cystic nephroma and multinodular goiter, with additional involvement of soft tissue and central nervous system tumors reported with variable frequency [1,4]. A rare phenotype termed GLOW syndrome (global developmental delay, lung cysts, overgrowth, and Wilms tumor) has been described in individuals with mosaic missense variants affecting the RNase IIIb domain of DICER1 [2]. Functional studies in these cases have demonstrated activation of the PI3K/AKT/mTOR pathway, suggesting a potential mechanism underlying the observed overgrowth phenotype [7].

In this report, we present a 16-year-old male patient whose clinical manifestations significantly overlap but also differ from those of GLOW syndrome. His clinical picture is notable for generalized overgrowth, mild developmental delay, pronounced macrocephaly, the presence of a multinodular goiter, and the detection of a unilateral pulmonary cyst. Furthermore, genetic testing identified a pathogenic germline nonsense variant in the DICER1 gene.

## 2. Materials and Methods

Clinical evaluation included detailed anamnesis and physical examination, with assessment of auxological parameters and bone age (BA). Laboratory investigations comprised routine biochemistry, analysis of autoantibodies, hormonal profiling, and tumor markers. Cytogenetic evaluation was performed by karyogram analysis. Imaging studies included computed tomography (CT) and ultrasound examinations. The patient was monitored through regular clinical and laboratory follow-up. The Reynolds Intellectual Assessment Scales, Second edition (RIAS-2) was used to assess the participant’s development at 17 years of age. The questionnaire is conducted in native language for the proband (Bulgarian).

### Genetic Analysis

Genomic DNA was extracted from peripheral blood of the proband. Targeted regions were enriched using the Roche KAPA HyperPlus hybrid capture-based protocol and sequenced on an Illumina MiSeq platform at the Department of Pathology, Jewish General Hospital (Montreal, QC, Canada). Only parental DNA was extracted from peripheral blood and analyzed by Sanger sequencing in the Molecular Genetics Laboratory, McGill University Health Center Research Institute (Montreal, QC, Canada). Sequence analysis was performed using the DICER1 reference transcript NM_177438.2, and variant nomenclature followed Human Genome Variation Society recommendations based on this RefSeq transcript, where nucleotide c.1 corresponds to the A of the ATG translation initiation codon.

Parental and patient informed consent was obtained in accordance with ethical research standards.

## 3. Case Report

The proband is the only child of healthy, non-consanguineous parents. Pregnancy and delivery were uneventful. He was delivered at term by elective Cesarean section due to obstetric indications. At birth, his anthropometric parameters already suggested an overgrowth phenotype, with a birth weight of 4.2 kg (+2.02 SDS) and a length of 52 cm (+1.08 SDS). Head circumference was disproportionately increased—42 cm (+3.8 SDS), consistent with macrocephaly. Apgar scores were normal, and there were no immediate neonatal complications. During early childhood, developmental delay became evident. Motor milestones were largely within the expected range, with independent sitting at 6 months and walking at 13 months. However, speech development was notably delayed, with first words emerging only after the age of 2 years. The proband received speech therapy during early school age group (up to 9 years), with subsequent improvement in language skills. No episodes of seizures, hypotonia, or significant feeding difficulties were reported, though his parents described mild clumsiness and delayed coordination in early years. He attended mainstream school but experienced learning difficulties, particularly in language-related tasks, requiring educational support. Cognitive assessment indicated low-average cognitive functioning. A formal neurological examination was normal at the age of 14 years. No formal assessment for autism spectrum disorder or behavioral abnormalities was undertaken, and brain MRI was not performed. The family history was remarkable for a strong predisposition to both benign and malignant tumors (Figure 1). On the maternal side, his mother had a history of thyroid nodules and a benign uterine tumor diagnosed in early adulthood. His maternal grandmother and grandfather were affected by breast cancer and lung cancer, respectively. On the paternal side, the grandmother had been diagnosed with colon cancer and endometrial cancer. No other family members were known to have congenital anomalies, developmental delay, or overgrowth syndromes, and there was no reported history of consanguinity.

The proband remained in good health until the age of 11 years, when he was referred to pediatric endocrinology for evaluation of tall stature (+2.62 SDS), with the clinical suspicion of Klinefelter syndrome (Figure 2). Serial anthropometric measurements demonstrated persistent tall stature for family as well as for the general population throughout adolescence with height SDS as follows: 14 years and 8 months, +2.6 SDS; and 16 years and 9 months, +1.54 SDS. At that time, clinical examination revealed macrocephaly, with a head circumference of 56 cm (+1.97 SDS). Distinct craniofacial features included a broad forehead, depressed nasal bridge, and hypodontia. Growth velocity was increased but proportionate, and body mass index was within the overweight (Figure 3). Laboratory investigations, including thyroid function tests, metabolic panel, and growth hormone axis assessment, were all within normal limits. Bone age was appropriate for chronological age, and chromosomal analysis by karyotyping showed a normal 46, XY male.

At age 14 years, following a mild COVID-19 infection, a chest radiograph was performed as a part of the routine evaluation. Unexpectedly, it revealed a large right-sided cystic lesion. Further characterization by chest CT confirmed the presence of a large, thin-walled, air-filled cyst occupying the right hemithorax (Figure 4). There was no evidence of solid components, hemorrhage, or invasive features.

Concurrent thyroid ultrasound performed at that time identified a multinodular goiter with multiple small nodules (Figure 5), though serum thyroid-stimulating hormone (TSH), free thyroxine (FT4), and triiodothyronine (T3) levels remained within normal range. As part of longitudinal follow-up, an abdominal ultrasound performed in 2024 revealed new findings of hepatic steatosis and mild splenomegaly, without associated symptoms or evidence of portal hypertension. The pancreas and kidneys appeared normal, and no adrenal masses or renal cysts were identified. A summary of relevant laboratory findings is provided in Table 1.

Given the strong family history and the constellation of clinical features—including tall stature, macrocephaly, thyroid nodules, pulmonary cyst and delayed neurocognitive development, all implying to the possibility of GLOW syndrome—molecular testing was pursued. A heterozygous nonsense variant in *DICER1*, c.1525C>T, p.(Arg509Ter), was identified through next-generation sequencing. This variant is predicted to result in premature truncation of the protein, leading to loss of functional domains. Pathogenicity was supported by classification criteria from the American College of Medical Genetics and Genomics (ACMG). The variant was confirmed by Sanger sequencing, which also detected the same alteration in the patient’s mother, thereby demonstrating maternal transmission. The variant was classified as pathogenic according to the laboratory report and is listed in ClinVar (Variation ID: 254288). Following confirmation of the *DICER1* mutation, the patient was evaluated by a thoracic surgeon, and after discussion of the findings and the potential risk of type II pleuropulmonary blastoma (PPB), a decision was made to pursue conservative management with annual CT surveillance. With regard to neurocognitive development, assessment using the Reynolds Intellectual Assessment Scales, Second Edition (RIAS-2), was performed at the age of 17 years. The results showed a Verbal Intelligence Index (VIX) of 96, within the average range, a Nonverbal Intelligence Index (NIX) of 81, corresponding to the low-average-to-borderline range. The Composite Intelligence Index of 87, indicating low-average overall cognitive functioning.

## 4. Discussion

This patient’s presentation contributes to the expanding phenotypic spectrum of DICER1 syndrome and underscores its notable clinical variability and clinical overlap with GLOW syndrome. The absence of clinical and imaging markers for malignant neoplasms in both the patient and his mother—who harbors the identical pathogenic variant and remains free of malignancy at the 46 years of age—illustrates the reduced penetrance and variable expressivity characteristic of this condition. Germline DICER1 pathogenic variants are now recognized as having relatively low penetrance; approximately 5.3% of carriers develop a neoplasm before age 10, rising to ~31.5% by age 60, which is still a considerably lower age than in the general population [8]. Many carriers remain asymptomatic into adulthood, reflecting incomplete penetrance [9].

The mechanism of DICER1-related tumorigenesis often follows a two-step model: a germline loss-of-function variant is followed by a somatic “hotspot” mutation in the RNase IIIb domain. Haploinsufficiency may also play a role, as complete loss of DICER1 function is incompatible with life [1,4]. Mosaic hotspot mutations, by contrast, can cause GLOW syndrome—characterized by global developmental delay, lung cysts, overgrowth, and Wilms tumor—highlighting the range of phenotypic consequences depending on mutation type and timing [1]. In the presented patient, genetic analysis revealed a germline heterozygous nonsense variant in *DICER1* (c.1525C>T, p.Arg509Ter), introducing a premature stop codon consistent with a loss-of-function first-hit mutation. While previously reported GLOW syndrome cases have been associated with missense hotspot mutations in the RNase IIIb domain that alter miRNA processing, the current case involves a truncating germline variant that nonetheless exhibits overlapping phenotypic features, including tall stature, macrocephaly, thyroid nodules, and pulmonary cysts. These findings expand the phenotypic spectrum associated with *DICER1* loss-of-function variants and suggest that partial GLOW-like manifestations may also occur in individuals harboring non-hotspot *DICER1* mutations.

Given the possibility of unaffected carriers and variable risk trajectories, tailored surveillance remains essential. The consensus protocol established by the SIOPE Host Genome and CanGene-CanVar working groups recommends a minimum program consisting of annual clinical examinations from birth to age of 20 years, chest radiography and renal ultrasound every six months from birth to 6 years, and thyroid ultrasound every three years from age 8 to 40 [4]. GeneReviews proposes similar measures, including chest imaging every 4–6 months until age 8 and annually thereafter until age 12; thyroid ultrasound every 3–5 years beginning at age 8; renal ultrasound every six months until age 8 and then annually until age 12; pelvic ultrasound in females every 6–12 months; and annual ophthalmologic evaluation for ciliary body medulloepithelioma between ages 3 and 10 [9].

Our patient’s case most closely resembles reports of the so-called “DICER1 syndrome plus” phenotype, combining dysmorphic features, lung cysts, and multinodular goiter, but lacking Wilms tumor, cystic nephroma, or fetal lung adenocarcinoma [2,10]. This raises the possibility that mutations within the RNase III domains may underlie extended syndromic presentations. Notably, the variant observed in the presented family has previously been reported in only four female individuals, all with ovarian sex cord–stromal tumors between 14 and 28 years of age [11]. In contrast, no such presentation was observed in our proband or his mother, although further screening for Sertoli–Leydig cell tumor remains warranted.

Pleuropulmonary blastoma (PPB) is the hallmark pediatric tumor of DICER1 syndrome, with >70% of cases associated with germline variants. Lung cysts in adolescents and adults with DICER1 variants are increasingly recognized. In one recent cohort of 110 individuals, 38% of those over 12 years of age had lung cysts detectable on CT imaging [12]. Management of such lesions depends on age, clinical presentation, size, and radiographic features. Our patient’s lung cyst was incidentally detected at age 14 following COVID-19 infection and has remained radiologically stable; thus, we elected for annual CT surveillance. In contrast, Tutor et al. described a complicated cyst presenting with hemoperitoneum, highlighting the importance of individualized management [13]. This contrasts with reported cases of complicated cysts requiring urgent intervention, which emphasizes the importance of individualized case-based management.

Thyroid abnormalities are common in DICER1 carriers though lifetime risk of malignancy remains below 15%. In one study, cumulative thyroidectomy rates for multinodular goiter by age 40 reached 75% in women and 17% in men, compared with 8% and 0% in controls [14]. Familial multinodular goiter, with or without SLCT, has been documented in several cohorts [15,16,17]. In the presented family, multinodular goiter remains stable under biannual ultrasound surveillance, further underscoring the variable expression of this trait.

DICER1 syndrome is associated with a characteristic spectrum of predominantly pediatric and young adult tumors, including pleuropulmonary blastoma, cystic nephroma, ovarian Sertoli–Leydig cell tumor, thyroid lesions (such as multinodular goiter and differentiated thyroid carcinoma), and rare central nervous system tumors such as pituitary blastoma [11,14,15,16]. In contrast, the family history in the present case includes common adult-onset malignancies (e.g., breast, colorectal, and endometrial cancer), which are not part of the recognized DICER1 tumor spectrum and are frequently encountered in the general population. The mother has a history of common malignancies and is also a carrier of the same DICER1 variant. However, a key limitation is that genetic testing and detailed clinical evaluation were not available for other family members beyond the parents, which limits the ability to fully assess segregation and broader familial cancer patterns.

Beyond neoplasia, non-tumor manifestations such as macrocephaly, tall stature, and developmental delay are increasingly recognized as part of the DICER1 spectrum. Khan et al. reported overgrowth and macrocephaly in 42% of mutation carriers [18]. Developmental delay has also been described in patients with 14q32 deletions encompassing DICER1 [19], in GLOW syndrome [2], and in “DICER1 syndrome plus” [10]. Our proband shares these features, lending further support to the emerging view that DICER1 mutations can result in a neurodevelopmental syndrome in addition to tumor predisposition. To our knowledge, the previously reported carriers of the germline DICER1 c.1525C>T (p.Arg509Ter) variant did not have documented overgrowth or neurodevelopmental abnormalities [11]. Consequently, the phenotype observed in our patient may represent phenotypic expansion or variable expressivity of this variant; however, definitive genotype–phenotype correlations cannot be established based on a single case. Hepatic steatosis identified on abdominal ultrasound in the proband has no established association with DICER1 syndrome in the current literature. Accordingly, this finding is considered incidental and is unlikely to be related to the underlying DICER1 variant. Alternative non-syndromic and metabolic etiologies should therefore be considered in the clinical interpretation.

A key limitation of our case is the lack of comprehensive genomic and epigenomic characterization, which contributes to the uncertainty of the variant’s interpretation. Moreover, existing literature provides limited evidence linking overgrowth or tall stature to germline DICER1 mutations occurring outside the RNase IIIb domain, further constraining phenotype–genotype correlations. Consequently, we have tempered our conclusions and state that the association with GLOW syndrome is based on the clinical phenotype, rather than direct functional evidence of mTOR pathway activation. Additional limitations of this study should be acknowledged. First, the single-case design precludes any definitive genotype–phenotype correlations or causal inferences regarding the observed clinical features. Second, functional validation studies were not performed, including RNA-based analyses, microRNA (miRNA) profiling, or assessment of downstream pathways such as mTOR signaling, which limits mechanistic interpretation of the identified variant. Third, familial genetic testing was incomplete, as molecular confirmation was only available for parents, while other relatives were not tested, restricting full evaluation of segregation patterns. Finally, longitudinal standardized neurodevelopmental assessments were not available, which limits the ability to objectively characterize developmental trajectory over time. These limitations should be considered when interpreting the findings of this report.

In summary, the absence of clinical and imaging marker for malignancy in both our proband and the mother, despite the presence of the previously described pathogenic variant of the DICER1 gene supports the hypothesis of its reduced penetrance. Aggressive interventions are therefore not warranted in asymptomatic carriers. Instead, adherence to structured surveillance protocols and shared decision-making regarding intensity of follow-up represent the most appropriate strategy [4,20]. The proposed surveillance protocol includes a minimum recommended program as well as an extended option for consideration. The minimum program consists of clinical and imaging-based surveillance measures for which the SIOPE Host Genome Working Group and CanGene-CanVar consortium consider that the potential benefits outweigh the associated harms. These recommendations are primarily based on expert consensus or majority agreement, albeit with variable levels of supporting evidence, with the exception of annual neck palpation, which is supported by limited evidence and restricted expert consensus. Annual clinical review is recommended from birth until 20 years of age to assess for signs and symptoms suggestive of DICER1-associated tumors, alongside ongoing patient and family education to maintain vigilance. Clinical examinations should ideally be coordinated by a designated clinician responsible for both assessment and the organization and interpretation of imaging surveillance, to ensure continuity of care and appropriate follow-up. It is further suggested that surveillance in carriers of pathogenic DICER1 variants from birth to 8 years of age is coordinated by pediatric oncology services, given the broad spectrum of tumor types occurring in early childhood. Beyond the age of 8 years, follow-up may transition to (pediatric) endocrinology, as the minimum surveillance program in this age group is primarily focused on thyroid surveillance. An additional challenge in surveillance design is the potential for harm. These include overtreatment (e.g., surgical intervention for asymptomatic benign lesions detected incidentally), need for sedation in young children during imaging procedures, cumulative radiation exposure, and the psychosocial burden associated with repeated investigations and false-positive findings [20].

## 5. Conclusions

The present case adds to the expanding phenotypic spectrum of DICER1 syndrome and highlights its clinical overlap with GLOW syndrome and “DICER 1 syndrome plus”. The absence of malignant manifestations in both the proband and his mother, despite the presence of a pathogenic DICER1 variant, further illustrates the reduced penetrance and variable expressivity within the affected families.

This case also supports the growing recognition that *DICER1* syndrome is not solely a tumor predisposition disorder but may also include neurodevelopmental and syndromic manifestations. Careful clinical evaluation, appropriate genetic counseling, and adherence to structured surveillance protocols, combined with individualized risk assessment and shared decision-making, remain the cornerstones of management. Further longitudinal studies and functional investigations are needed to refine genotype–phenotype correlations, optimize surveillance strategies, and improve our understanding of the natural history of *DICER1*-associated disorders.

## Figures and Tables

**Figure 1:**
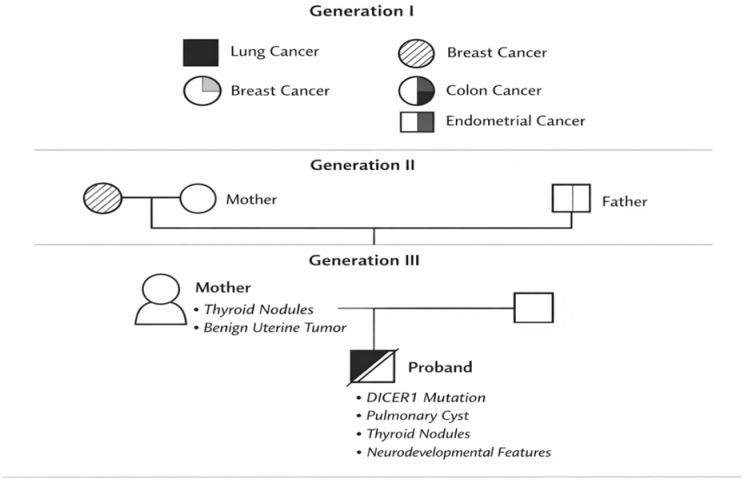
Pedigree of the family.

**Figure 2:**
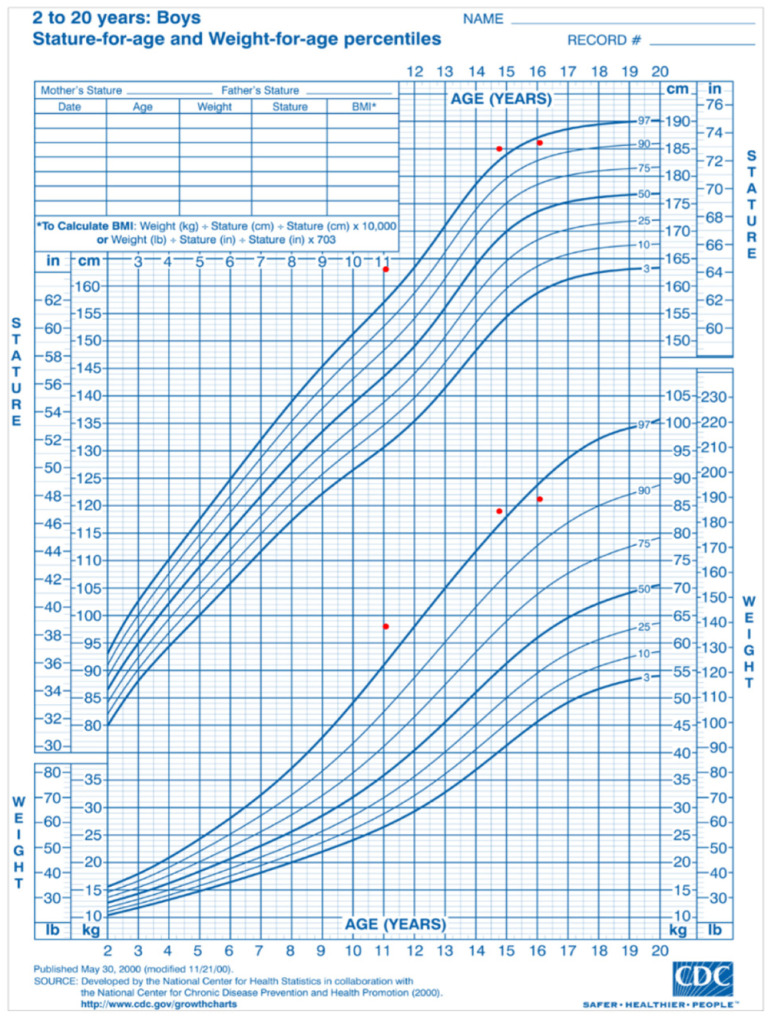
Growth pattern (Midparental height 175 cm, −0.01 SDS).

**Figure 3:**
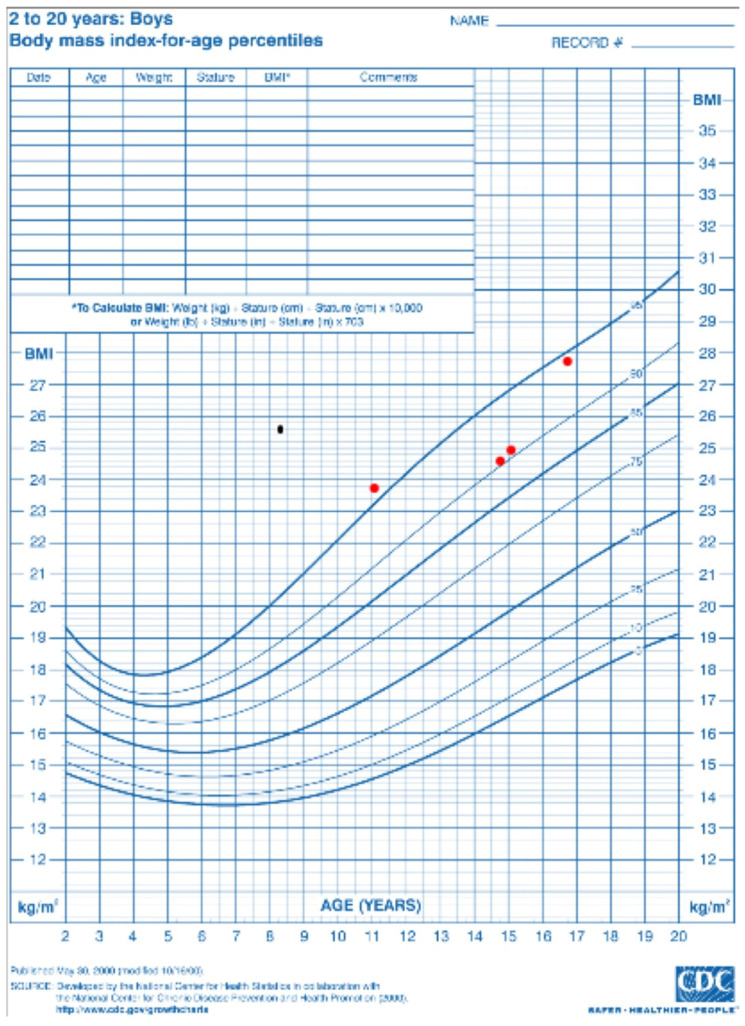
BMI data varying +1.58 SDS to +1.68 SDS.

**Figure 4:**
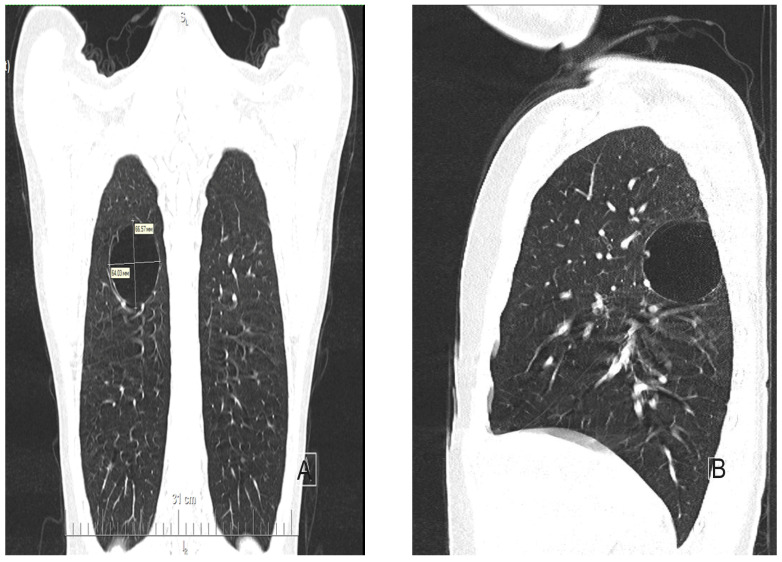
CT of the thorax. (**A**): CT from 2023 demonstrates a thin-walled pulmonary cyst in the right lung, located in the sixth pulmonary segment, measuring 66 × 66.6 mm. (**B**)—Sagittal cross of the cyst.

**Figure 5:**
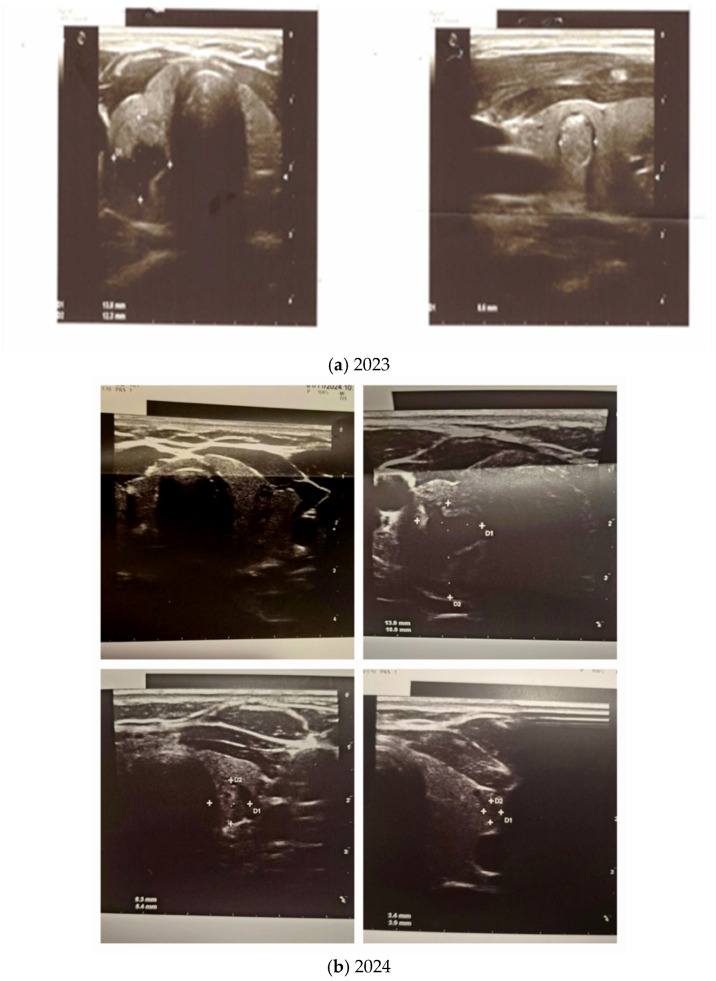
Ultrasound of thyroid gland in two consecutive years ((**a**) 2023; (**b**) 2024). (**a**) Mixed nodule, partially solid with a central cystic/unechogenic component in the right lobe (13.8 × 12.3 mm). In the left lobe, a solitary solid nodule is noted in the mid-portion, measuring 8.6 mm. (**b**) Cystic nodule in the mid-to-posterior region of the right thyroid lobe (13 × 18 mm) with an unechogenic component, occupying almost the entire lobe. A small paraesophageal structure (8.3 × 8.4 × 10 mm) and an adjacent hypoechoic area (~3 mm) are noted in the lower 1/3 of the lobe.

**Table 1:** Laboratory data follow-up.

Parameter	Reference Range	2019	2022	2023	2024
TSH (mIU/mL)	0.51–4.3	1.28	1.38	1.38	1.7
FT3 (pmol/L)	3.93–7.7				5.76
FT4 (pmol/L)	12.6–21	14.2	18.37	17.24	17.6
Anti-TPO (U/mL)	0–34				8.66/negative
Anti-TG (uIU/mL)	0–95				16.43/negative
TG (ng/mL)	3.5–77				22.53
LH (mIU/mL)		1.88	4.42		5.98
FSH (mIU/mL)		2.15	3.07		3.58
Testosterone (nmol/L)		6.24			18.6
Prolactin (uIU/mL)	53–360		273	223	303
Growth hormone (ng/mL)	0.05–3.0		4.93	0.524	<0.05
IGF-1 (ng/mL)	173–414	526	340	302	266
IGF BP3 (ug/mL)	3.4–9.5		6.1	6.09	
Ratio IGF/BP3			0.056	0.0495	
Alpha-fetoprotein (ng/mL)	0.6–6			1.8	1.96
hCG (mIU/mL)	0–2.7			2.7	1.9

## Data Availability

The data that support the findings of this study are available from the corresponding authors upon reasonable request.

## Abbreviations

The following abbreviations are used in this manuscript:

PPB	Pleuropulmonary blastoma
SLCT	Sertoli–Leydig cell tumor
MNG	Multinodular goiter
GLOW syndrome	Global developmental delay, Lung cyst, Overgrowth, Wilms tumor
BA	Bone age
CT	Computed tomography
